# Leptin at gender-specific concentrations does not affect glucose transport, expression of glucose transporters and leptin receptors in human lymphocytes

**DOI:** 10.1007/s12020-014-0435-3

**Published:** 2014-10-12

**Authors:** Monika Pliszka, Bożenna Oleszczak, Leszek Szablewski

**Affiliations:** Chair of General Biology and Parasitology, Center for Biostructure Research, Medical University of Warsaw, 5 Chalubinskiego Str., 02-004 Warsaw, Poland

**Keywords:** Leptin, Leptin receptor, Deoxy-d-glucose, Lymphocytes, Glucose transporters

## Abstract

Leptin shows pleiotropic effects in organisms including an important role in the regulation of glucose homeostasis. Elevated serum leptin, particularly in obese individuals, is a warning sign of energy imbalance, hyperinsulinemia, insulin resistance and other metabolic risk factors that are strongly associated with type 2 diabetes. Obesity is also related to a higher rate of infections and immune function deterioration may in part ensue from decreased glucose uptake as the main energy source for lymphocytes. The aim of this study was to investigate the effect of physiologic and low pathophysiologic gender-specific leptin concentration found in lean and obese subjects on glucose transport, the expression of glucose transporters and leptin receptors in human peripheral blood lymphocytes. Isolated lymphocytes were incubated with human leptin at gender-specific concentrations observed in normal weight and obese subjects. Glucose uptake in lymphocytes was determined using nonmetabolizable radiolabeled deoxy-d-glucose. The expression of GLUT1, 3, 4 and leptin receptors was investigated using methods of immunocytochemistry and flow cytometry. Leptin at concentrations used in the study does not change glucose transport into lymphocytes and seems to have no influence on the expression of glucose transporters and leptin receptors. Further studies are necessary to address the relationship between leptin, glucose transport and the lymphocytes’ function in obesity.

## Introduction

Leptin is a hormone that plays a key role in metabolic processes and immune functions. Plasma circulating leptin levels directly correlate to total body fat mass and increase proportionally in obesity [9]. Serum leptin level depends on gender and leptin is found to be from 2.35 to 3.5-fold higher in females [18, 19] than in males matched for body mass [43]. Elevated leptin levels coexist not only with obesity but also with diabetes and metabolic syndrome [7], the states that are connected with impaired immunity [2, 13].

Leptin has been recognized as an important factor for modulating the immune responses. Leptin receptors (ObR) are expressed in several cell types of innate and adaptive immunity including lymphocytes [34]. Studies on animals and humans with complete leptin deficiency showed markedly reduced numbers of lymphocytes with impaired humoral responses [5]. There is also an evidence that leptin has effects on innate and acquired immune responses [11, 23]. Leptin stimulates the secretion of other proinflammatory cytokines in the innate immune system such as IL-1, IL-6 and TNF-α [25], and increases phagocytic activity of macrophages [29]. In relation to the acquired immune system, leptin treatment enhances proliferation and suppresses apoptosis of T lymphocytes [26, 40]. Recent studies have indicated that leptin also activates human peripheral blood B cells to induce secretion of IL-6, IL-10, and TNF-α [1].

Though obesity and its associated complications like type 2 diabetes or metabolic syndrome are well defined, little is known about the impact of obesity on immune functions per se. Obesity is associated with decreased immunocompetence [24] and immunity deterioration is related to the grade of obesity [32]. Obese subjects are highly susceptible to infectious diseases, some types of cancer [32, 37] and have lower lymphoproliferative responses to mitogens [8]. Obesity also leads to increased levels of leptin creating a proinflammatory state which may affect lymphocytes number, function [10] and the activation of immune cells [12]. Several obesity-associated changes such as leptin resistance, hyperinsulinemia, excessive inflammation, and altered glucose metabolism which are required for the functionality of lymphocytes could affect the immune response [2]. Moriguchi et al. [35] proposed that the decreased lymphoproliferative response observed in obese rats may be, in part, due to decreased glucose uptake as the main energy source for lymphocytes and may be associated with the decreased expression of Glut1. Unfortunately there is a lack of research in the area concerning human lymphocytes. Moreover it remains unclear whether data from rodent studies also apply to humans [30].

Lymphocytes are cells that have high metabolism with extreme dependency on glucose as a source of energy [36]. Some studies indicate that lymphocytes require glucose metabolism for normal survival and function [28]. Lymphocytes have been found to express GLUT1, GLUT3 and GLUT6 that are primarily responsible for glucose transport in the resting state [14, 31]. In lymphocytes of diabetic patients and women with polycystic ovary syndrome as well as in lymphocytes incubated in high glucose in medium GLUT4 is also expressed [38, 39, 49]. Many studies have found that T cells increase glucose uptake during an immune response [33]. Regulation of T cell glucose uptake and Glut1 is critical, as low glucose prevent appropriate T cell responses [20] and can suppress immunity [54].

It seems that the mechanism affecting impaired immunity in obesity is not fully understood. For that reason more research is necessary to understand what factors are contributing to reduced immunocompetence in obesity state. We chose leptin from among several factors in obesity to determine its role in glucose transport and expression of glucose transporters in lymphocytes. In our study we used the cells derived from lean subjects to exclude the influence of other obesity-associated factors that may affect lymphocytes functions.

It is clear that leptin acts as a hormone that links metabolic processes and immune functions. Based on findings that leptin exerts its effects on lymphocytes and in some types of cells it can stimulate transport of glucose it seems interesting that leptin may be a factor that impact glucose transport into human lymphocytes.

The aim of this study was to investigate the effect of physiologic and low pathophysiologic gender-specific leptin concentration found in lean and obese subjects on glucose transport, the expression of glucose transporters and leptin receptors in human peripheral blood lymphocytes.

## Materials and methods

### Subjects

It has been shown that age and obesity are accompanied by leptin resistance [41], so the study was undertaken in 100 young, normal weight subjects (50/50 W/M). Written informed consent was obtained from all participants after approval by the Bioethical Committee of the Medical University of Warsaw. Subjects were excluded if they had a history of any serious illness (including diabetes in the family) and other that may affect insulin sensitivity or if they used medications during 3 months preceding the study. The basic characteristics of the examined subjects are presented in Table 1. Anthropometric measurements were done on the day of blood samples collection. Blood glucose was assayed in capillary using a glucometer (Contour, Bayer). The study was carried out according to the principles of the Helsinki Declaration (updated in Edinburgh).

**Table 1:** Characteristics of subjects participated in the study

	Women	Men
*n*	50	50
Age (years)	23.4 ± 2.3	24.2 ± 1.6
Height (cm)	168.4 ± 4.6	184.2 ± 3.6
Weight (kg)	62.9 ± 3.5	78.2 ± 4.7
BMI (kg/m^2^)	22.3 ± 1.3	23.1 ± 1.4
Waist (cm)	80.1 ± 4.2	98.4 ± 2.5
Glucose (mg/dl)	91.7 ± 1.1	93.3 ± 1.9

### Isolation of lymphocytes from the blood

Blood samples were collected in the morning, after an overnight fast, into tubes with heparin. Lymphocytes were isolated within next 2 h by means of centrifugation in Histopaque 1077 (Sigma-Aldrich) gradient, according to the manufacturers’ instructions. The isolated cells were rinsed three times by centrifugation (1,800 rpm, 10 min, 24  C) in PBS. After this procedure cell density in 1 ml of PBS was counted using the Bürker chamber.

### Incubation of lymphocytes with human leptin

Lymphocytes were incubated for 24 h with or without (control sample) human recombinant leptin (R&D Systems) at physiologic (4 ng/ml for men and 8 ng/ml for women) and low pathophysiologic (8 ng/ml for men and 32 ng/ml for women) concentrations. The concentrations of leptin used in the present study were based on studies in which serum leptin level were measured in lean (BMI 20–25) and obese (BMI 30–35) men and women respectively [17, 27, 42].

Lymphocytes were incubated in sterile Petri dishes (2.5 × 10^6^ cells in each dish) in DMEM no glucose medium (Gibco) supplemented with 10 % fetal bovine serum, 100 μg/mL of streptomycin and 80 mg/dL of glucose (equivalent of normoglycemia) in a humidified 37 °C, 5 % CO_2_ incubator. After incubation, cells were rinsed from the medium and suspended in 1 mL of transport solution (20 mM Hepes, 150 mM NaCl, 5 mM KCl, 5 mM MgSO_4_, 1.2 mM KH_2_PO_4_, 25 mM CaCl_2_, 2 mM pyruvic acid, pH 7.4) [21].

### Lymphocytes viability control test

The test was conducted to verify the survival of the cells during the experiment. To 290 µL of cellular suspension, 1.5 µL of deoxy-d-glucose 2-[^3^H(G)] (8.0 Ci/mmol; Perkin Elmer) was added. After 60 min, 1 % solution of trypan blue was added at 1:1 volume ratio. The number of dead lymphocytes in a sample of 500 cells was counted using a light microscope.

### Intracellular transport of deoxy-d-glucose into lymphocytes

Glucose uptake in lymphocytes was determined using nonmetabolizable radiolabeled hexose–2-deoxy-d-glucose, considered the gold standard for examining glucose transport [51]. The study was carried out according to the method described by Kaliman et al. [21] with a partial modification [48].

To 290 µL of cellular suspension (about 3 × 10^5^ lymphocytes) 1.5 µL of deoxy-d-glucose 2-[^3^H(G)] (8.0 Ci/mmol; Perkin Elmer) and 7.5 µL of PBS were added. The non-specific uptake of tritium-labeled deoxy-d-glucose was assessed using 7.5 µL of cold “stop solution” (50 mmol/L of d-glucose in PBS, 4 °C) instead of PBS solution.

In order to investigate the dynamics of deoxy-d-glucose uptake by lymphocytes, the incubation time with isotope equaled 15, 30, and 60 min. After this time, 200 µL of cold “stop solution” was added to each probe. Lymphocytes (about 3 × 10^5^ cells) were lysed by adding 50 µL of lysing solution (1.1 mmol/L NaOH; 0.1 % SDS). 24 h later 25 µL of lysed cells (about 1.5 × 10^5^ lymphocytes) was taken to evaluate the deoxy-d-glucose uptake.

The amount of deoxy-d-glucose uptake was measured with a liquid scintillation counter (Microbeta Trilux Wallac). Radioactivity was evaluated in *cpm* (curie per minute). The label uptake was assessed basing on results of total label accumulated at given time minus the nonspecific uptake of deoxy-d-glucose.

### Immunocytochemistry

Lymphocytes (about 5 × 10^4^ from each group) were dried and endogenic peroxidase was blocked by adding 200 μL of 3 % H2O2 solution. Then lymphocytes were placed in blocking buffer (1 % bovine serum albumin in PBS) with 2 % goat serum (Sigma). After 30 min mouse monoclonal antibody (1:200) against extracellular domain of human leptin receptor (R&D Systems) was added and then horseradish peroxidase-conjugated goat anti-mouse IgG (1:1000) (Chemicon International Inc. Ca). The negative control sample (for exclusion of non-specific binding of antibodies) consisted of lymphocytes incubated without the first antibody. The same procedure was used in the case of glucose transporters. The antibodies used were rabbit polyclonal antibody aimed against intracellular C-terminus of human GLUT1, GLUT3 and GLUT4 (1:100) and horseradish peroxidase-conjugated goat anti-rabbit IgG (1:2000) (Chemicon International Inc. Ca).

The antigen–antibody complex was visualized using DAB according to the manufacturers’ instructions (Sigma-Aldrich). The presence of investigated proteins was assessed using a light microscope (800×).

### Flow cytometry

Lymphocytes (about 3 × 10^5^) from each probe were washed in buffer for FACS (PBS without Mg^2+^ and Ca^2+^ with the addition of 2 % fetal bovine serum and 0.002 % sodium azide) by centrifugation (1,300 rpm, 10 min, 4 °C). Cells were permeabilized using 100 μL of Perm2 (Becton–Dickinson) for 10 min. After washing, cells were suspended in 100 μL of buffer for FACS and placed on ice. Samples were incubated for 60 min with 2 μL of polyclonal rabbit antibodies appropriate with: anti-GLUT1, anti-GLUT3 and anti-GLUT4 (Chemicon International Inc. Ca) synthetic peptides corresponding to the C-terminus of human GLUT at a 1:50 dilution. The secondary antibody was 3 μL of swine anti-rabbit IgG-FITC (Dako Cytomation). Cells were incubated on ice for 30 min in the dark. The control sample (negative) was incubated only with the secondary antibody. The supernatant was removed, and 0.5 mL of washing buffer for FACS with 1 % formaldehyde were added.

For investigating the expression of leptin receptors in plasma membrane, the same procedure was used as in the case of GLUT proteins, excluding permeabilization. As the first antibody, 2 μL (500 μg/mL) of mouse monoclonal antibody against extracellular domain of human leptin receptor (R&D Systems) was used. Phycoeritrin-conjugated goat anti-mouse IgG (1:200) (R&D Systems) was used as the secondary antibody.

The samples were analyzed using the FACS Calibur flow cytometer (Becton-Dickinson) fitted with an argon laser (wavelength, 488 nm) and CellQuest software.

### Statistical analysis

The results were presented as mean ± SD for males and females separately. Statistical analysis was performed by two-way ANOVA with replication for continuous variables. A *P* value <0.05 was considered statistically significant.

## Results

### Lymphocyte viability control test

The test demonstrated that the applied research method did not have a significant impact on lymphocyte survival. A similar percentage of dead lymphocytes (about 5 %) were observed in individual samples.

### Deoxy-d-glucose uptake by lymphocytes

**Fig. 1:**
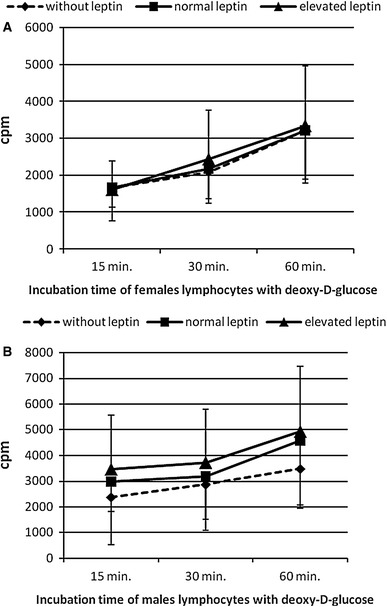
The intensity of deoxy-d-glucose uptake by females lymphocytes (**a**) and males lymphocytes (**b**) in dependence on leptin concentrations in incubating medium. The results (*n* = 20 for each gender) were presented as mean ± SD. Statistical analysis was performed by two-way ANOVA with replication. Differences between control and leptin-treated lymphocytes were not statistically significant. Normal leptin refers to physiologic level: 4 ng/ml for men and 8 ng/ml for women. Elevated leptin refers to low pathophysiologic level: 8 ng/ml for men and 32 ng/ml for women

In analyzed time points (15, 30 and 60 min.) *cpm* value increased which meant that after passage of time the amount of deoxy-d-glucose transported into cells was bigger. So deoxy-d-glucose uptake studied in females and males lymphocytes has a clearly growing tendency. However, leptin treatment has not caused statistically significant differences in glucose transport in comparison to control lymphocytes. Figure 1 presents the deoxy-d-glucose uptake by females and males lymphocytes properly in dependence on leptin concentrations in the incubating medium.

### Immunocytochemistry

In all investigated females and males samples, irrespective of leptin concentrations, single positive colored cells have been observed suggesting the presence of leptin receptors (ObR) in lymphocytes. Negative control did not return color reaction in any of the samples. It is to note that many lymphocytes did not show positive reaction with antibody against ObR. When investigating GLUT proteins, positive cells have been found in all of the samples, independently of leptin concentrations. However, no differences in color intensity between lymphocytes incubated in different leptin concentrations have been found in males and females, respectively.

**Fig. 2:**
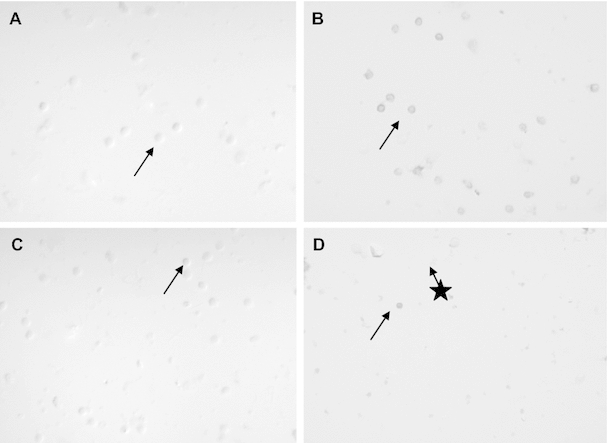
Immunocytochemical detection of GLUT4 and leptin receptors in lymphocytes. **a** Negative control for females lymphocytes incubated without the first antibody. The *arrow* shows an exemplary negative cell. **b** Females lymphocytes incubated in elevated leptin concentration showed positive reaction against GLUT4. The *arrow* shows an exemplary lymphocyte with positive reaction against GLUT4. **c** Negative control for males lymphocytes incubated without the first antibody. The *arrow* shows an exemplary negative cell. **d** Males lymphocytes incubated in elevated leptin concentration showed positive reaction against leptin receptor. The *arrow* shows an lymphocyte with positive reaction against leptin receptor. The *arrow* with a star shows an lymphocyte without positive reaction against leptin receptor

All lymphocytes that were colored, showed similar intensity of immunological reaction. That is why in Fig. 2 we show only an exemplary result of immunocytochemical detection of GLUT4 and leptin receptor.

### Flow cytometry

**Fig. 3:**
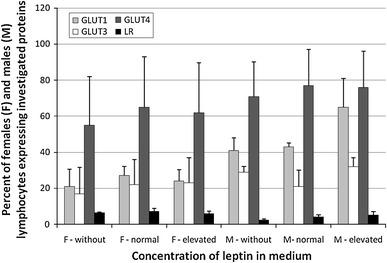
Percent of females and males lymphocytes showing expression of GLUT proteins and leptin receptors. The results (*n* = 20 for each gender) were presented as mean ± SD. Statistical analysis was performed by two-way ANOVA with replication. Differences between control and leptin-treated lymphocytes were not statistically significant Normal leptin refers to physiologic level: 4 ng/ml for men and 8 ng/ml for women. Elevated leptin refers to low pathophysiologic level: 8 ng/ml for men and 32 ng/ml for women

**Fig. 4:**
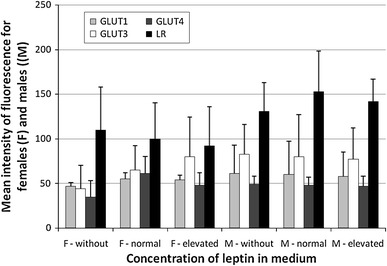
Mean intensity of fluorescence of particular GLUT proteins and leptin receptor (LR) in females and males lymphocytes. The results (*n* = 20 for each gender) were presented as mean ± SD. Statistical analysis was performed by two-way ANOVA with replication. Differences between control and leptin-treated lymphocytes were not statistically significant. Normal leptin refers to physiologic level: 4 ng/ml for men and 8 ng/ml for women. Elevated leptin refers to low pathophysiologic level: 8 ng/ml for men and 32 ng/ml for women

**Fig. 5:**
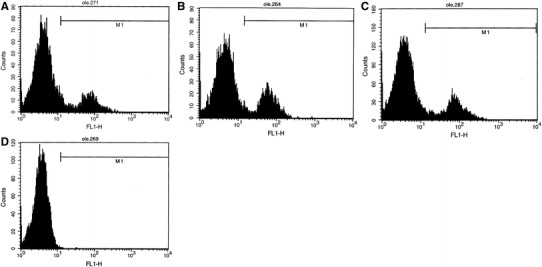
Representative histograms of GLUT1 from flow cytometry analysis of females samples incubated in different leptin concentration. The first antibody used was polyclonal rabbit antibody against GLUT1. The secondary antibody was swine anti-rabbit IgG-FITC. **a** control without leptin; **b** normal leptin concentration; **c** elevated leptin concentration; **d** control sample (negative) without antibody against GLUT1

In order to obtain quantitative values that described expression of investigated proteins, the flow cytometry analysis was used. In females only a few percent of lymphocytes showed the presence of leptin receptors (Fig. 3), irrespectively of leptin concentration and differences between particular samples were not statistically significant. Incubation with different leptin concentration had also no impact on the percent of cells expressed GLUT transporters (Fig. 3). Mean intensity of fluorescence (MIF) makes possible the quantitative comparison of investigated proteins in cells. When particular GLUT and lepin receptors are reported as MIF, no differences have been found irrespective of leptin concentrations (Fig. 4). Similarly, in male lymphocytes different leptin concentrations had no effect on the percent of cells which expressed leptin receptors and particular GLUT transporters (Fig. 3) as well as on MIF values (Fig. 4). Figure 5 shows representative histograms of GLUT1 from flow cytometry analysis of females samples incubated in different leptin concentration.

The obtained result suggests that leptin in our experiment does not affect the quantity of GLUT proteins in the whole cells and leptin receptors in the cell membrane.

## Discussion

The current study has been the first to investigate the effect of low pathophysiologic leptin concentration found in obese subjects on glucose transport, the expression of glucose transporters and leptin receptors in human lymphocytes. The results showed that independent of leptin concentration used, there was no effect on glucose uptake as well as on glucose transporters and leptin receptors in lymphocytes of lean subjects.

According to Sahu [44] in hypothalamus leptin and insulin share a similar PI3K (phosphatidylinositol-3-kinase) intracellular signaling pathway that is thought to play a role in glucose uptake and metabolism [16]. In lymphocytes the PI3K pathway has been show to promote Glut1 cell surface trafficking and activity [50]. In the absence of these signals Glut1 remains intracellular and may restrict glucose uptake [20].

The results of our study have demonstrated that elevated leptin concentration typical for obese subjects did not change deoxy-d-glucose transport into lymphocytes. It appears that this fact is in opposition to the findings of Moriguchi et al. [35] who suggested that obesity coexisted with increased leptin concentration in rats may decrease glucose uptake by lymphocytes. On the other hand many researchers have shown the effect of leptin on glucose uptake in different peripheral tissues. In rat and mouse skeletal muscle cells leptin is able to inhibit or stimulate glucose uptake in vitro [3, 4, 6, 22, 47]. Sweeney et al. [47] showed the effect of leptin on glucose uptake in rat muscle cells but leptin level in their experiments was very high (100 nM = 1,600 ng/mL), several times higher than in obese human subjects (with BMI from 30 to 35) and in comparison to those used in our study.

In the present study the lack of differences in glucose transport between control and leptin-treated lymphocytes may result from the lack of change in the percent of lymphocytes expressing investigated GLUT transporters. Mean intensity of fluorescence which is a sign of amount of GLUT protein in the cell also shows no difference in all samples.

The obesity state is connected with impaired glucose metabolism as well as the role of GLUTs. Different leptin concentrations used in our study, typical for normal weight and obese subjects, had no effect on the expression of GLUTs in lymphocytes. However, according to Saucillo et al. [46] leptin acts directly on activated T cells to allow glucose uptake and metabolic reprogramming. Lymphocytes in our experiments were not stimulated with any additional factors apart from leptin and probably behaved as resting cells. It seems that leptin alone in concentration used in our study is not a sufficient factor for the activation of lymphocytes. The studies of Saucillo et al. [46] indicated that the ability of leptin to upregulate T cell Glut1 expression and glucose metabolism was observed only in activated lymphocytes and resting T cells did not increase glucose metabolism following leptin therapy.

The effective leptin action on immune system cells requires the expression of specific receptors on the surface of cells [45]; Zhao et al. [53]. Górska and Wąsik [15] observed the expression of leptin receptors on B and T cells derived from bone marrow and peripheral blood in children. This study has revealed a dozen of ObR positive B and T cells in peripheral blood. A small number of cells expressing leptin receptors was indicated by Zarkesh-Esfahani et al. [52]. They found leptin receptors only in peripheral lymphocytes B, but in lymphocytes T leptin receptors were not present. The percentage of ObR positive lymphocytes in our study was similar to the results of Zarkesh-Esfahani et al. [52]. This fact may also suggest that ObR positive cells in our study belong to B cells.

Leptin receptors in the cell membrane show similar mean intensity of fluorescence in lymphocytes incubated without leptin in comparison to those incubated in different leptin concentrations. This suggests that the amount of protein in the cell membrane was the same in all ObR positive lymphocytes. It is a possibility that resting lymphocytes have a constant amount of leptin receptors on their cell surface and leptin at concentrations used in our experiments exerts no effect. In the present paper we failed to find the effect of different leptin concentrations on the expression of leptin receptors in lymphocytes. Probably this results from the fact that investigated lymphocytes were not activated.

In conclusion, we have demonstrated that leptin as a single factor had no effect on the intensity of glucose transport into human lymphocytes across genders. The lack of leptin action on glucose transport may be caused by the lack of differences in the expression of glucose transporters in cells incubated in low and elevated leptin concentration. Leptin concentrations used in the present study have also no impact on the percentage of ObR positive lymphocytes and the amount of leptin receptors on the surface of cells. Further studies including obese patients and/or high pathophysiologic and pharmacologic leptin concentration will be necessary to address the relationship between leptin, glucose transport, and lymphocyte function in obesity.
